# Multi-modal Dataset of a Polycrystalline Metallic Material: 3D Microstructure and Deformation Fields

**DOI:** 10.1038/s41597-022-01525-w

**Published:** 2022-08-01

**Authors:** J. C. Stinville, J. M. Hestroffer, M. A. Charpagne, A. T. Polonsky, M. P. Echlin, C. J. Torbet, V. Valle, K. E. Nygren, M. P. Miller, O. Klaas, A. Loghin, I. J. Beyerlein, T. M. Pollock

**Affiliations:** 1 University of Illinois at Urbana-Champaign, Urbana, 61801 USA; 2 University of California Santa Barbara, Santa Barbara, 93106-5050 USA; 3 Materials Mechanics & Tribology Department, Sandia National Laboratories, Albuquerque, NM 87185 USA; 4 Institut PPRIME, Université de Poitiers, CNRS, ENSMA, UPR 3346, 86962 Chasseneuil Cedex, France; 5 Cornell High Energy Synchrotron Source, Sibley School of Mechanical and Aerospace Engineering, Cornell University, Ithaca, NY 14853 USA; 6 Simmetrix, Clifton Park, NY 12065 USA

**Keywords:** Mechanical properties, Metals and alloys

## Abstract

The development of high-fidelity mechanical property prediction models for the design of polycrystalline materials relies on large volumes of microstructural feature data. Concurrently, at these same scales, the deformation fields that develop during mechanical loading can be highly heterogeneous. Spatially correlated measurements of 3D microstructure and the ensuing deformation fields at the micro-scale would provide highly valuable insight into the relationship between microstructure and macroscopic mechanical response. They would also provide direct validation for numerical simulations that can guide and speed up the design of new materials and microstructures. However, to date, such data have been rare. Here, a one-of-a-kind, multi-modal dataset is presented that combines recent state-of-the-art experimental developments in 3D tomography and high-resolution deformation field measurements.

## Abstract (summary)

**Table:** 

Measurement(s)	3D grain structure and grain orientations • Surface deformation fields
Technology Type(s)	Electron backscatter diffraction (EBSD) • Scanning electron microscope (SEM) • TriBeam microscope • high resolution digital image correlation
Factor Type(s)	applied macroscopic strain • crystallographic orientation
Sample Characteristic - Organism	nickel superalloy Inconel 718
Sample Characteristic - Environment	mechanical deformation

## Background & Summary

During mechanical loading, polycrystalline materials, such as the nickel-based superalloy investigated here, develop irreversible plastic deformation that can manifest in the formation of slip bands. Consequently, slip traces develop and are observed at the free surface of deformed specimens, with each slip trace associated with a local surface step. This step is produced by dislocations emerging from the free surface during plastic deformation after gliding along crystallographic planes in the bulk^1^.

The systematic investigation of slip as a function of the microstructure helps to better identify the relevant parameters that control the plastic deformation flow. These connections provide useful insight into the design and prediction of mechanical properties in structural components. The quantification of plastic localization by slip (slip localization) is challenging in polycrystalline metals. Nanometer-scale spatial resolution is required to experimentally observe individual slip events during loading that can occur over tens to hundreds of atomic planes. At the same time, larger, millimeter-scale fields are also required to capture the material response over statistically-representative populations of microstructural (grain) configurations. In addition, it has recently been demonstrated that the three-dimensional (3D) grain structure is important for identifying microstructural configurations that are preferential locations for slip^2^. While the grain structure at a free surface is useful to identify some of the microstructural features of interest, an understanding of the complex grain boundary network in the bulk is necessary to identify all of them. As a consequence, a representative volume element (RVE) for deformation of polycrystalline metallic materials must encompass the 3D grain structure over a large field of view with accurate representation of the deformation fields at the sub-grain scale.

Recent advances in scanning electron microscopy (SEM), 3D tomography and multi-modal data analysis have provided unique opportunities to measure and analyze, in a reasonable amount of time, slip activity over near mm^2^ -scaled fields of view as a function of the 3D microstructure. In the present paper, slip localization measurements during monotonic loading have been performed by high resolution digital image correlation (HR-DIC) on a face centered cubic nickel-based Inconel 718 superalloy. Quantitative measurements were performed over large regions of interest on this multi-modal dataset for statistically significant correlations between the microstructure and slip event locations and amplitudes. The grain structure at the sample surface was captured by conventional electron backscatter diffraction (EBSD) measurements. However, the full subsurface 3D grain structure was collected by TriBeam serial sectioning^3^. Advanced data merging tools were used to produce a distortion-free multi-modal dataset. This dataset is one of a kind and will provide the scientific community a new means of investigating the effect of two-dimensional (2D) and 3D microstructure on slip localization, and a benchmark dataset for model development and validation. All raw measurement data are provided. In addition, the final reconstructions of the dataset, in the form of both voxelized and meshed 3D structures, are also provided to allow direct comparison between experimental data and computational modeling. Finally, the 3D volume mesh built from the voxelized 3D microstructural data fully renders the grain and grain boundary morphology and is prepared for finite element crystal plasticity analysis.

## Methods

### Material and mechanical testing

Wrought Inconel 718 (In718) (nominal composition in wt% Ni - 0.56%Al - 17.31%Fe - 0.14%Co - 17.97%Cr - 5.4%Nb - Ta - 1.00%Ti - 0.023%C - 0.0062%N) was subjected to a 30 minute annealing treatment at 1050 °*C* followed by water quenching, producing a grain size distribution centered at 62 *μ*m with a nearly random texture. A two-step precipitation hardening treatment consisting of 8 hours at 720 °*C* then 8 hours at 620 °*C* was conducted to form hardening *γ*' and *γ*″ precipitates^4^.

**Fig. 1:**
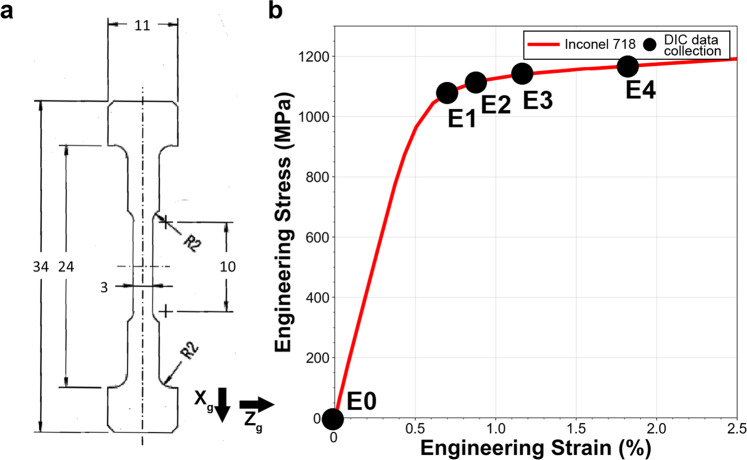
Monotonic loading of the *in-situ* (SEM) macroscopic specimen. (**a**) Specimen geometry used for *in-situ* mechanical testing. The dimensions are given in millimeters. (**b**) Engineering stress-strain curve for the investigated nickel-based superalloy Inconel 718. The black dots display the stress/strain at which the HR-DIC measurements were performed.

Tensile testing was performed at room temperature at a quasi-static strain rate using a custom *in-situ* ± 5000 N stage within a ThermoFisher Versa3D microscope on a flat dogbone-shaped specimen with a gauge section of 1 × 3 mm^2^. The geometry of the tensile specimen is provided in Fig. 1(a). The tensile specimen was cut by wire electrical discharge machining, mechanically mirror polished using abrasive papers and diamond suspensions, and then chemo-mechanically polished using a suspension of 0.04 *μm* colloidal silica particles. The tensile test was interrupted at macroscopic plastic strain levels of 0.17%, 0.32%, 0.61% and 1.26% for collection of high resolution images for HR-DIC measurements while loaded. The macroscopic strain was measured *in-situ* using fiducial markers located at both ends of the gauge length. The engineering stress-strain curve for this material is shown in Fig. 1(b) and the corresponding data are provided. The (X_*g*_, Y_*g*_, Z_*g*_) coordinate system (g here standing for global) is used throughout the entire manuscript and is consistent between figures.

### High-resolution digital image correlation

**Fig. 2:**
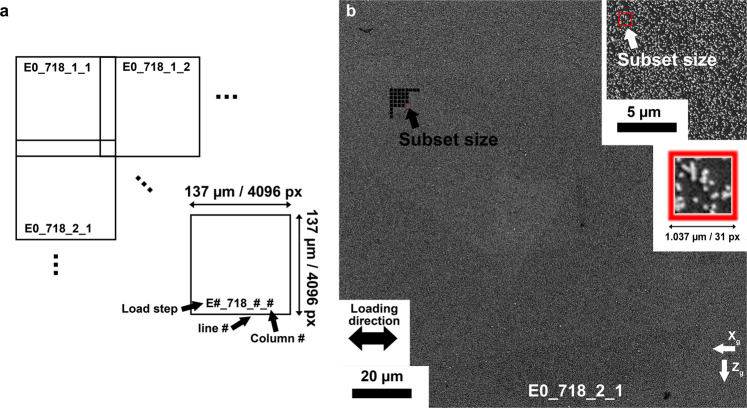
Scanning electron microscope images for high-resolution measurement of deformation fields. (**a**) Notation of the SEM images for HR-DIC measurements. (**b**) A SEM micrograph of one region of interest from the HR-DIC experiment, imaged at a horizontal field width of 137 μm; enlarged images of the speckle pattern are contained in the inset image. The black and red boxes indicate the subset size of 31 × 31 pixels (1036.86 nm × 1036.86 nm).

A gold nanoparticle speckle pattern with average particle size of 60 nm was deposited on the sample surface for DIC measurements, following the procedure developed by Kammers *et al*.^5^. SEM image sets were acquired from the middle of the gauge length before loading and under load following the guidelines of Kammers and Daly^5,6^ and Stinville *et al*.^7^. A National Instruments^TM^ scan controller and acquisition system (DAQ) was used to control beam scanning in the ThermoFisher microscope. This custom beam scanner removes the SEM beam defects associated with some microscope scan generators^7,8^. Tiles of 8 × 8 SEM images, before and after deformation, with an image overlap of 15% were collected. All images were converted to 8-bit *tiff* format before digital image correlation. They are provided for the different loading steps. Their labeling is explained in Fig. 2(a). Each image was acquired with a dwell time of 20 μs, a pixel resolution of 4096 × 4096 and a horizontal field width of 137 μm. Consequently, each pixel has a size of 33.45 nm.

Regions of about 1 × 1 mm^2^ were investigated for the Inconel 718 nickel-based superalloy. DIC calculations are performed on these series of images and the results are merged using a pixel resolution merging procedure found elsewhere^9^. A subset size of 31 × 31 pixels (1036.86 nm × 1036.86 nm) with a step size of 3 pixels (100.34 nm) was used for the DIC measurements. DIC was performed using the Heaviside-DIC method^10,11^. The resulting strain fields *ε*_*xx*_ are calculated using strain windows of 3 pixels (100.34 nm), where *ε*_*xx*_ is the strain field along the loading direction, X (same axis as X_*g*_). The *ε*_*xx*_ strain field is provided for the entire investigated region and for each loading steps. For better visualization of the slip events, the strain field *ε*_*xx*_ was also processed using a decay filter of 4 pixels (401.37 nm). In all DIC strain fields, a single pixel represents three pixels (100.34 nm) in the SEM images.

**Fig. 3:**
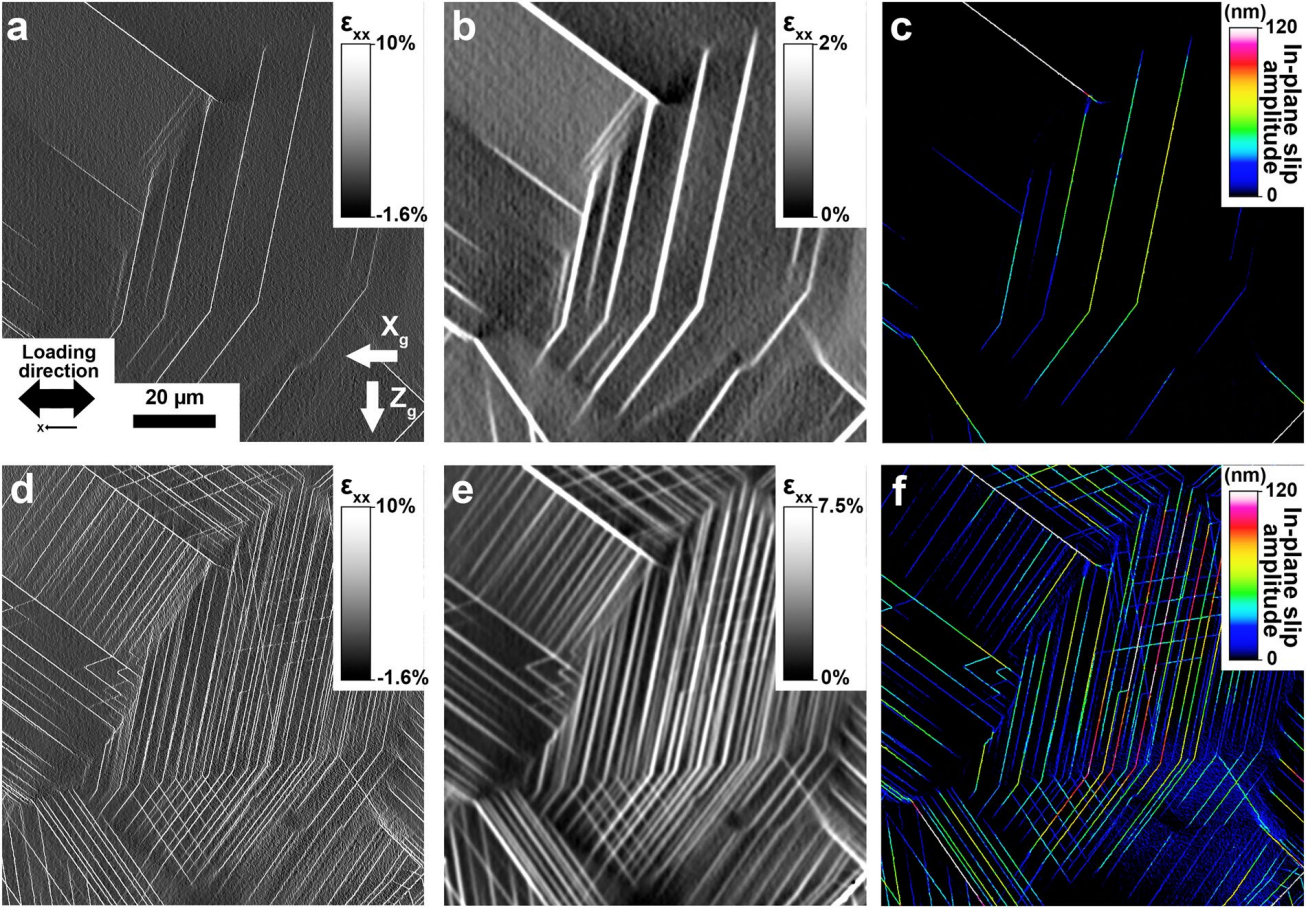
Sub-grain deformation field during monotonic tensile loading. DIC fields obtained from the Heaviside-DIC method after deformation at macroscopic plastic strain levels of 0.17% (**a**–**c**) and 1.26% (**d**–**f**) for a reduced region of interest. (**a**,**d**) The *ε*_*xx*_ strain field along the loading direction obtained from DIC with a spatial resolution of 100.34 nm. (**b**,**e**) Averaged strain field *ε*_*xx*_ along the loading direction obtained from DIC, with a spatial resolution of 401.37 nm. The resolution was reduced for better visualization of the slip events. (**c,f**) The intensity of slip events obtained by the Heaviside-DIC method.

Correlation between the deformed and undeformed images, given by the Heaviside-DIC method, provides the displacement field induced by slip. Consequently, from the Heaviside-DIC method, the full in-plane description of the slip displacements can be obtained at every point in the HR-DIC map. The in-plane slip displacements represent the physical displacement in the plane of the surface produced by a slip event. More details on the Heaviside-DIC method can be found elsewhere^12^. The amplitude (norm) of slip is measured in nanometers for each measured point and is given in Fig. 3(c,f) for a reduced region of interest after plastic deformation at 0.17% (step labeled E1) and 1.26% (step labeled E4), respectively. The intensity of slip is obtained for each individual slip trace with high spatial (less than 33 nm) and amplitude resolution (less than 10 nm). The local direction of slip is also calculated, providing direct identification of the distinct slip systems (slip plane and slip direction), when correlated to the local crystal orientation^10^.

### Surface crystallographic orientation measurements

**Fig. 4:**
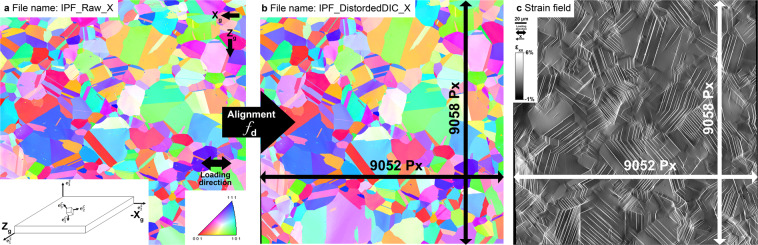
Correlative measurement between deformation field and surface microstructure. (**a**) Inverse pole figure map along the loading direction of the investigated region after unloading from macroscopic plastic deformation of 1.26%, obtained by 2D EBSD measurements. (**b**) The associated EBSD map after distortion correction to fit to the DIC fields obtained. (**c**) Averaged strain field *ε*_*xx*_ for the investigated region after 0.61% plastic deformation, corresponding to the loading step labeled E3.

EBSD analysis was performed to obtain the grain orientation at the DIC surface. These measurements were carried out with an OIM-Hikari XM4 detector using a step size of 0.7 μm. Diffraction patterns were acquired using an accelerating voltage of 20 kV, a 2 × 2 binning mode and a beam current of 0.2 nA. The EBSD map was acquired after deformation and after the unloading from step labeled E4 in Fig. 1(b). Crystallographic orientations can be described with three Euler angles, *ϕ*_1_, Φ, *ϕ*_2_. They represent the three elemental rotations required to describe the principle axes of the crystal with regard to the principle axes of the sample. In the present data, Bunge’s convention for these Euler angles is used. This is a so-called passive description as they constitute rotations needed to bring the sample coordinate frame into coincidence with the crystal coordinate frame. Let the axis (${e}_{1}^{S}$, ${e}_{2}^{S}$, ${e}_{3}^{S}$) and (${e}_{1}^{C}$, ${e}_{2}^{C}$, ${e}_{3}^{C}$) describe the specimen and crystal bases respectively. In the Bunge convention, the set of three Euler angles correspond to three sequential rotations, starting with a rotation (*ϕ*_1_) about the ${e}_{3}^{S}$ axis followed by a rotation (Φ) about the new ${e}_{1}^{S}$ axis, and followed by a third rotation (*ϕ*_2_) about the new ${e}_{3}^{S}$ axis again. The angles *ϕ*_1_ and *ϕ*_2_ range from 0 to 2 *π* and Φ ranges from 0 to *π*. The different axes are provided in the insert in Fig. 4(a). Figure 4(a) shows the inverse pole figure map along the loading direction X_*g*_ (horizontal) based on the raw orientation data. The EBSD data were then cleaned using a neighbor orientation correlation filter with a 5° grain tolerance angle followed by a grain dilatation filter with a 5° tolerance. Figure 4(b) shows the orientation map, also in inverse pole figure colors along the loading direction, after applying an alignment procedure described by Charpagne *et al*.^13^. This procedure aims at matching the EBSD data, which suffers from inherent, complex distortions, to the DIC full-field maps. The alignment procedure uses a two-dimensional polynomial function of degree 3, *f*_*d*_ (Eq. 1) calibrated by sets of control points selected from the EBSD and DIC maps, to align and re-sample EBSD data to the DIC maps with a resolution of one pixel. Here the aspect ratios of the grains are recovered from the inherent distortion induced by EBSD measurements. This map is also provided and overlaps directly with the DIC collection grid.

1 $$\begin{array}{l}f(x,y)=\left(\begin{array}{l}x{\prime} ={\sum }_{n=0}^{3}{\sum }_{k=0}^{n}{c}_{k,n-k}^{x}\,{x}^{k}{y}^{n-k}\\ y{\prime} ={\sum }_{n=0}^{3}{\sum }_{k=0}^{n}{c}_{k,n-k}^{y}{x}^{k}{y}^{n-k}\end{array}\right.\end{array}$$

Hereinafter, these EBSD measurements will be referred to as *surface EBSD measurements*, since they correspond to the crystallographic orientations on the DIC surface of the specimen.

### 3D Crystallographic orientation measurements

**Fig. 5:**
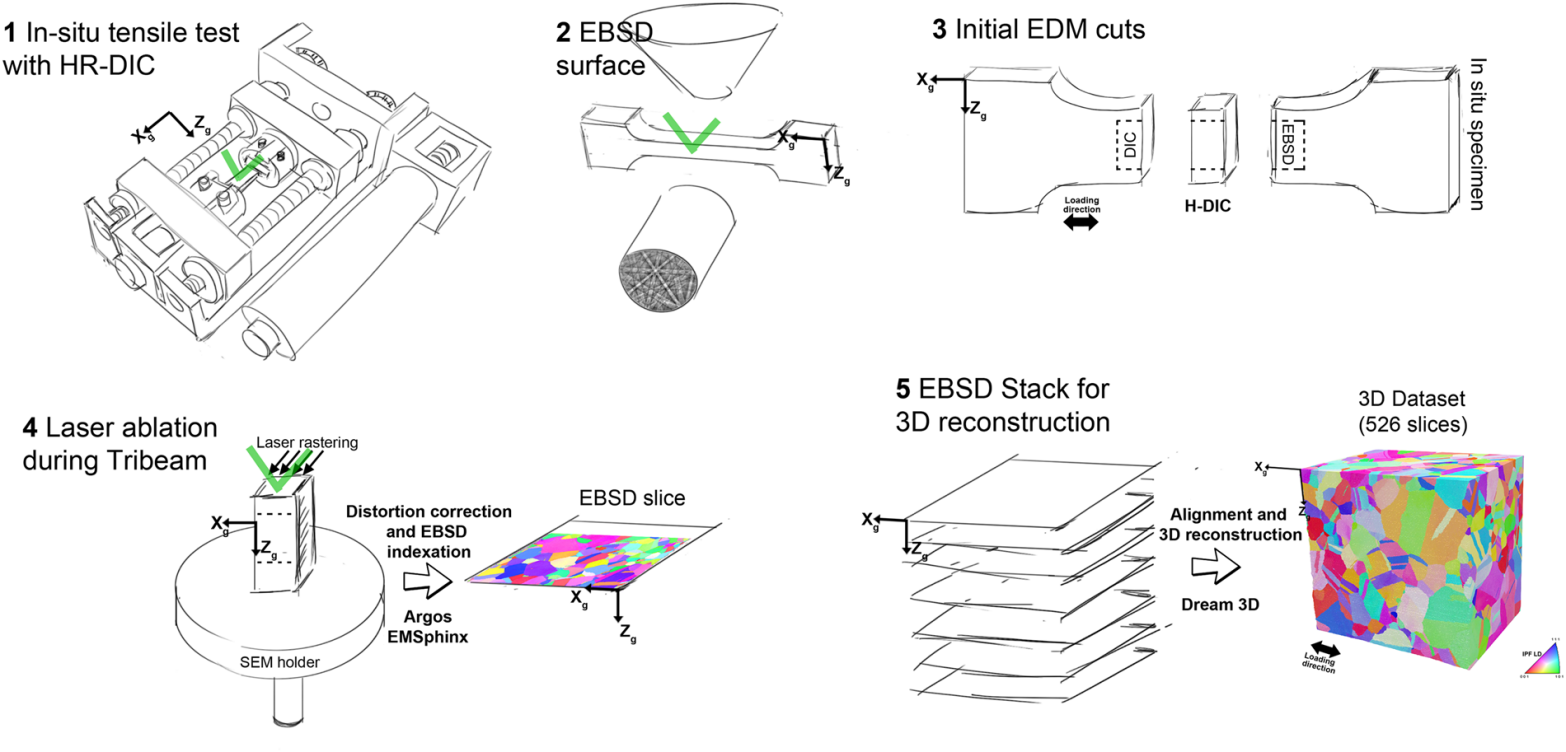
Experimental procedure for correlative measurements. **1** *In-situ* mechanical testing with HR-DIC. Tensile test performed inside the SEM using an *in-situ* stage. **2** The specimen is removed from the *in-situ* stage after loading and EBSD measurements are performed on the surface of the specimen. **3** Initial electrical discharge machining cuts were performed to prepare a pillar for TriBeam serial sectioning. **4** Laser ablation of the pillar with EBSD measurements collected between each slice. **5** Reconstruction of the 3D dataset. The green symbols indicate the direction of the incident electron beam during DIC or EBSD measurements.

The TriBeam system^3,14^ is used for the collection of orientation fields in 3D over a half cubic millimeter volume. A schematic of the overall experiment is provided in Fig. 5. After mechanical testing (Fig. 5(1)), the specimen is unloaded and surface EBSD measurements (see previous section) are performed on the surface of the specimen on the same region where the HR-DIC measurements were made ((Fig. 5(2)). Electrical discharge machining cuts were performed to prepare a pillar with optimal geometry for a TriBeam experiment, shown in Fig. 5(3). The pillar is laser ablated as depicted in Fig. 5(4) with a step size of 1 μm in Z_*g*_, the sectioning direction. Between each slice, EBSD measurements are collected with a step size of 1 μm  (X_*g*_,Y_*g*_) to form cubic voxels. Backscatter electron (BSE) images are collected from the top surface of the pillar (0° tilt) and secondary electron (SE) images are collected at a 30° tilt angle. BSE images were also collected every 30 slices for use with dataset stack alignment. A set of 526 slices was obtained during the experiment and reconstructed into a 3D dataset using the DREAM.3D software^15^ (Fig. 5(5)). Prior to reconstruction, each EBSD slice was aligned to match the corresponding BSE image using the procedure described above. The BSE images of the top surface of the pillar, collected 0° tilt were subjected to minimal distortions compared to the EBSD measurements, collected at 70° tilt^16^ and experiencing longer beam dwell times per pixel and hence larger beam drift. GrainIDs and FeatureIDs were defined during the reconstruction process using a miorientation-based grain segmentation tolerance of 5°. While grainIDs and FeatureIDs represent the same entities, grainIDs refer specifically to the grains in the meshed dataset.

**Fig. 6:**
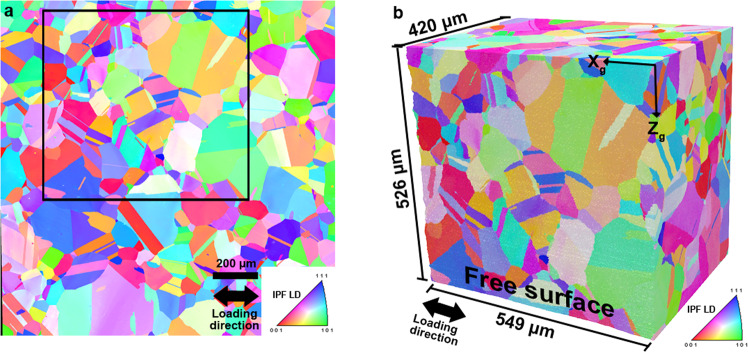
3D voxelized microstructure. (**a**) Inverse pole figure map referenced along the loading direction from surface EBSD measurements after specimen unloading from a macroscopic plastic deformation of 1.26%. (**b**) 3D reconstruction of the TriBeam experiment data from a region that contains a subset of the free surface displayed by the black box in **a**.

Figure 6 shows the final reconstruction of the 3D dataset from a subset of the specimen, where the free surface of the 3D dataset corresponds to the same surface where surface EBSD and HR-DIC measurements were collected. The black box in Fig. 6(a) highlights the location of the area mapped in 3D. A cubic voxel resolution of 1 μm was used to create a representation of the 3D grain structure of the investigated region i.e. a RVE. A comprehensive review on RVE classification, generation and usage is provided by Bargmann *et al*.^17^.

**Fig. 7:**
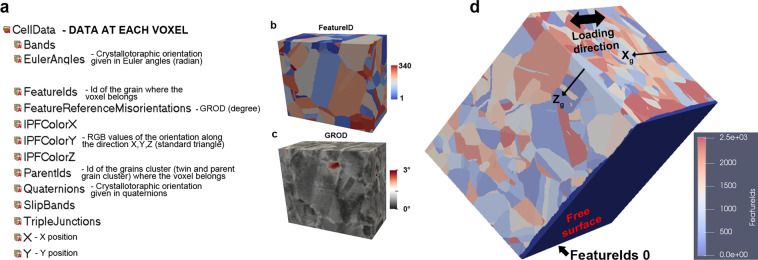
3D microstructure and information at each voxel. (**a**) TriBeam serial sectioning HDF data container file hierarchy. (**b**) FeatureID indicating the unique ID for each grain (parent grains or twins) for a subset of the 3D dataset. (**c**) Associated grain reference orientation deviation. (**d**) Dataset colored according to the FeatureIDs, with a unique ID for each grain for the 3D dataset. The FeatureIDsClean 0 indicates a surface layer on the surface where HR-DIC measurements were performed. This layer of unassigned voxels allows a perfect parallelepiped to facilitate simulation/calculations.

The information provided by TriBeam serial sectioning is detailed in Fig. 7. At each voxel, the crystallographic orientation as described by Euler angles is given and displayed in “EulerAngles” in the h5 file (Hierarchical Data Format version 5 - See Data Records section) of the 3D dataset. The convention for Euler angles representation is different than in the 2D EBSD data. Information on the conversion between the surface EBSD and 3D EBSD Euler angles is provided in the file “Transformation_EulerAngle_2d_3d.pptx”. Crystallographic orientations are also provided using unit quaternions in the h5 file. More information about the conventions used to store EBSD data and conversions between reference frames is in the following references^18,19^.

Figure 7 is colored according to the FeatureIDs, integer numbers that label continuous regions of voxels that have been identified as grains. A subset of the dataset with FeaturesIDs coloring is presented in Fig. 7(b). Grains (twin or parent grains) are identified as voxel regions that have been segmented using a tolerance angle of less than 5°. Clusters of twin-related grains^20^ and their associated parent features are indicated by an ID named ParentIDs. The FeatureIDs labeled as 0 are associated with the surface layer of the voxels with unassigned data at the free surface of the specimen. The reconstruction of the 3D dataset and alignment of EBSD slices (Fig. 5(5)) produce alignment errors from the stacking of slices that result in non perfectly flat surface (X-Z) in the two axes orthogonal to the slicing direction. The offsets in the free surface are filled with unassigned data to obtain a planar surface where DIC data was collected, as shown in Fig. 7(d). The other surfaces were simply cropped to obtain flat faces. All the contained array values at each voxel within the HDF h5 file are described in Fig. 7(a). This dataset includes the grain reference orientation deviation (GROD) calculated at each voxel, which is shown for a subset of the 3D dataset in Fig. 7(c). The value of the GROD, which is labeled as “FeatureReferenceMisorientation” in the .dream3d file, is the misorientation in degrees for each voxel in a grain, with respect to the average orientation of the grain.

### Correlative measurements: multi-modal data merging

**Fig. 8:**
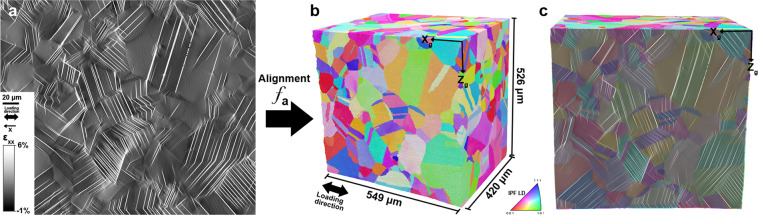
Correlative measurement between deformation field and 3D microstructure. (**a**) Subset of the HR-DIC *ε*_*xx*_ strain field that corresponds to the surface area where TriBeam serial sectioning has been performed. (**b**) The function *f*_*a*_ used to distort the DIC fields to match the surface of the 3D dataset. (**c**) The strain field *ε*_*xx*_ overlapped on the surface of the 3D dataset.

**Fig. 9:**
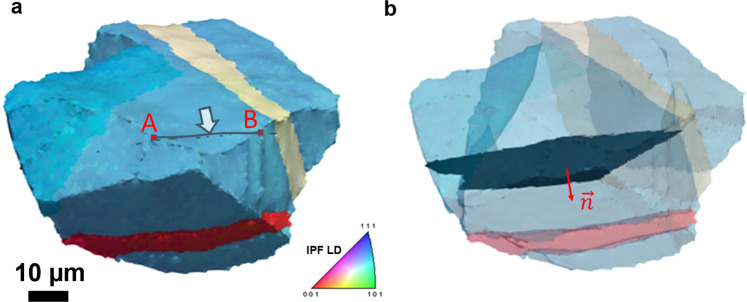
Reconstruction of a 3D slip band from a 2D slip trace. (**a**) A grain of interest and a slip trace that extends from the free surface into the subsurface bulk (highlighted with the arrow), where A and B are the coordinates of the endpoints of the slip trace. (**b**) 3D slip band reconstructed (indicated by normal $\overrightarrow{n}$) after multi-modal data merging and determination of the active plane. Reprinted from Charpagne *et al*.^2^ with permission from Elsevier.

The strain fields obtained from DIC corresponding to the investigated free surface of the 3D dataset are provided for the different loading steps. All fields have been aligned to fit the free surface of the 3D dataset as displayed in Fig. 8. The distortion between both datasets was modeled using a polynomial function of degree 3, described in Eq. (1). Individual slip traces were segmented from the DIC maps and indexed as individual features, using the iterative Hough transformation method presented in Charpagne *et al*.^13^. The location of each slip band in the 3D volume (coordinates of its endpoints on the (X_*g*_-Z_*g*_ surface), its inclination angle relative to the loading direction, its length and average in-plane slip intensity and direction are all calculated. With the assumption of slip on any of the twelve {111} <110> slip systems, the recombination of the slip trace inclination angle on the DIC surface with the local crystallographic orientation enables the determination of the active {111} slip plane. Since at least one point within each slip band and its crystallographic slip plane are known, all the slip bands can be projected and reconstructed in the 3D volume, as shown in Fig. 9. An angular tolerance was applied to obtain 1 voxel thick slip bands to accommodate the voxelized nature of the data. The voxels were assigned number IDs that refer to the slip bands they belong to, and a readily observable in the ‘SlipBands’ entry in the .dream3d file of the merged dataset. Individual slip bands can be viewed in Paraview as voxelized objects, or as ‘slice’ objects, as shown in Fig. 9.

Two mesh structures of the 3D dataset were created and detailed in the following sections. Mesh structures provide unique opportunities to perform finite element calculations to elucidate the effect of crystallographic orientation and/or 3D grain structure in metallic materials.

### Mesh generation with xtalmesh

**Fig. 10:**
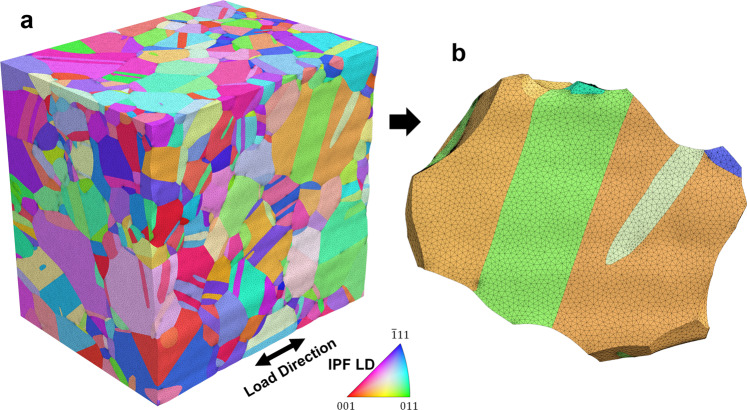
Mesh structure of the In718 dataset generated by XtalMesh. (**a**) Element sets are colored using the inverse pole-figure map according to the grains they represent. (**b**) A large parent grain is selected for closer inspection of the mesh.

One version of a mesh structure was created with XtalMesh^21^, and can be seen in Fig. 10. XtalMesh is used to create smooth representations of voxelized microstructures and leverages the state-of-the-art tetrahedralization algorithm fTetWild^22^ to generate an analysis-ready, boundary conforming tetrahedral mesh. The base workflow of XtalMesh was modified to better preserve the many small and thin features (mainly twins) of the Inconel 718 dataset from the effects of excessive smoothing (shrinkage and/or thinning).

XtalMesh works by first receiving as input the triangle surface geometry of all grains in the microstructure, in this case obtained via the *QuickSurfaceMesh* filter in DREAM3D^15^. Once received, XtalMesh smooths all grain boundary interfaces and triple junctions in a sequential manner using a constrained Laplacian smoothing algorithm. Details of the XtalMesh workflow and algorithms applied can be found in the original paper^21^. For this work, a customized workflow was developed whereby the XtalMesh smoothing operation was applied only to the parent grain surface mesh geometry. After smoothing, twinned regions of each parent grain were re-introduced into the parent grain surface mesh via the constructive solid geometry (CSG) technique^23^. The twin insertion process takes place as follows. For each twin, the convex hull is computed to create a smooth surface representation of the twin avoiding any possible shrinkage or thinning that could result from traditional smoothing operations. After this, two mesh Boolean operations are performed to define the twin and new parent grain mesh.

2 $${\bf{T}}={\bf{H}}\cup {\bf{P}}\,:=\left\{{\rm{x}}\in {{\mathbb{R}}}^{3}| {\rm{x}}\in {\bf{H}}\;{\rm{and}}\;{\bf{x}}\in {\bf{P}}\right\}$$

3 $${{\bf{P}}}_{{\rm{new}}}={\bf{P}}\backslash {\bf{T}}\,:=\left\{{\rm{x}}\in {{\mathbb{R}}}^{3}| {\rm{x}}\in {\bf{P}}\;{\rm{and}}\;{\rm{x}}\notin {\bf{T}}\right\}$$

**Fig. 11:**
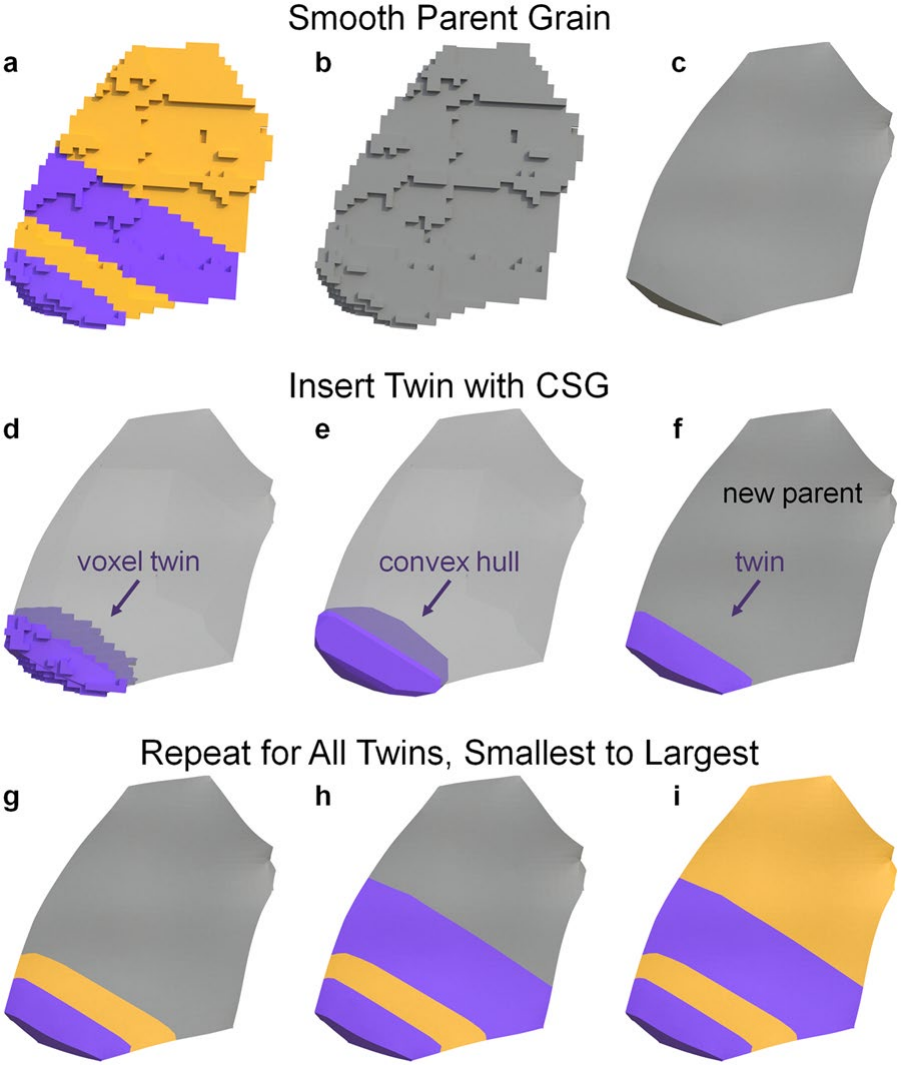
Diagram of the XtalMesh smoothing and twin insertion process for a single parent grain. The smoothing process ignores surface mesh geometry of all features (**a**) and considers only that of the parent grain (**b**), producing the smoothed parent grain mesh. (**c**) Twins are inserted back into the parent grain mesh by taking their now partially smoothed representations (**d**), computing their convex hull **e** and calculating the intersection with the parent grain mesh (**f**). (**g–i**) Insertion process repeats until all twins are inserted, in order of twin size, from smallest to largest.

**Fig. 12:**
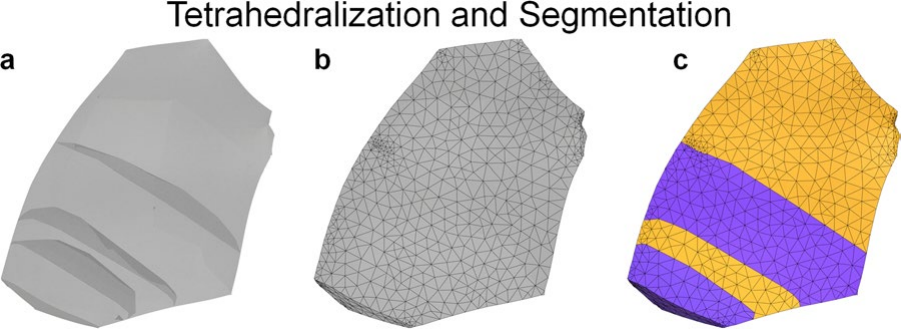
Diagram of the XtalMesh tetrahedralization and segmentation process for a single parent grain. (**a**) Surface mesh after all twin insertion is complete, input to fTetWild algorithm. (**b**) Volume mesh output of fTetWild, elements produced within grain surface meshes, but algorithm unaware of grain ID assignment. (**c**) Final mesh after segmenting elements according to grain IDs.

The twin mesh, **T**, is defined as the union of its convex hull, **H**, and parent grain mesh, **P** where *x* represents points that lie in real space ${{\mathbb{R}}}^{3}$. The new parent grain mesh, **P**_new_, is then given by the difference, \, between the previous parent grain mesh, **P**, and the inserted twin mesh, **T**. This twin insertion process is carried out for each twin in the order of smallest to largest based on the number of voxels. Figure 11 shows a diagram of the smoothing and twin insertion process for one chosen parent grain. After insertion of all twins was complete, tetrahedralization was performed on the resulting surface mesh of the entire microstructure using the fTetWild meshing algorithm^24^. Because the output of fTetWild is a tetrahedral mesh, with no grain ID information assigned to the elements, a segmentation procedure followed that assigned grain ID numbers to elements depending on which individual grain surface mesh they lie within. Figure 12 displays how this process works for the previously examined parent grain. The final mesh contains 19.9 million tetrahedral elements.

### Geometric reconstruction and mesh generation using simmetrix’ software suite

Accurate representation of the material structure at the mesoscale level in the finite element domain is one requirement to model grain-level deformation process and to attempt fulfilling any validation requirements. Existing geometric representation and mesh generation capabilities^25^ can close the technological gap in converting EBSD serial-sectioning stacks to high-definition volumetric meshes suitable for finite element analysis.

While it is possible to directly generate a mesh from a voxel dataset it is advantageous to introduce a geometric model, specifically a non-manifold boundary representation^26^ as an intermediate representation of the analysis domain^27^. Such a model provides an unambiguous representation of the analysis domain and provides a mechanism to associate information such as material properties in a manner that is independent of the mesh. Assigning meshing attributes on the topology of this model (e.g., interfaces between grains or the boundary of the domain) or based on geometry (mesh refinement in areas of interest) allows multiple meshes to be automatically generated from this representation^27^. To be able to build a valid and appropriate (based on the needs of the simulation) finite element model from a voxel dataset assembled from a serial sectioning EBSD measurement, various procedures to remove artifacts are required. This includes the elimination of small groups of disconnected voxels, and removing noise from the grain boundaries (e.g., through the use of erosion and dilation filters^28^). This process is followed by the elimination of physically undesirable voxel configurations (e.g., voxel clusters of the same material connecting at a single voxel corner) that could create singularities in the finite element solution. The resulting geometric model represents each grain as a region (volume) with geometric faces (surfaces) representing grain boundaries. Attributes attached to each region allow the user to retrieve the grain ID as it was defined in the originating DREAM3D^15^ dataset. At this stage, the face geometry still reflects the stair-stepped boundaries between the individual voxels, therefore a geometric based algorithm is used to create smooth geometric faces while preserving the overall shape of the grain boundaries. The resulting geometric model can be tagged with meshing and analysis attributes to generate a run-ready input deck for the finite element solver.

**Fig. 13:**
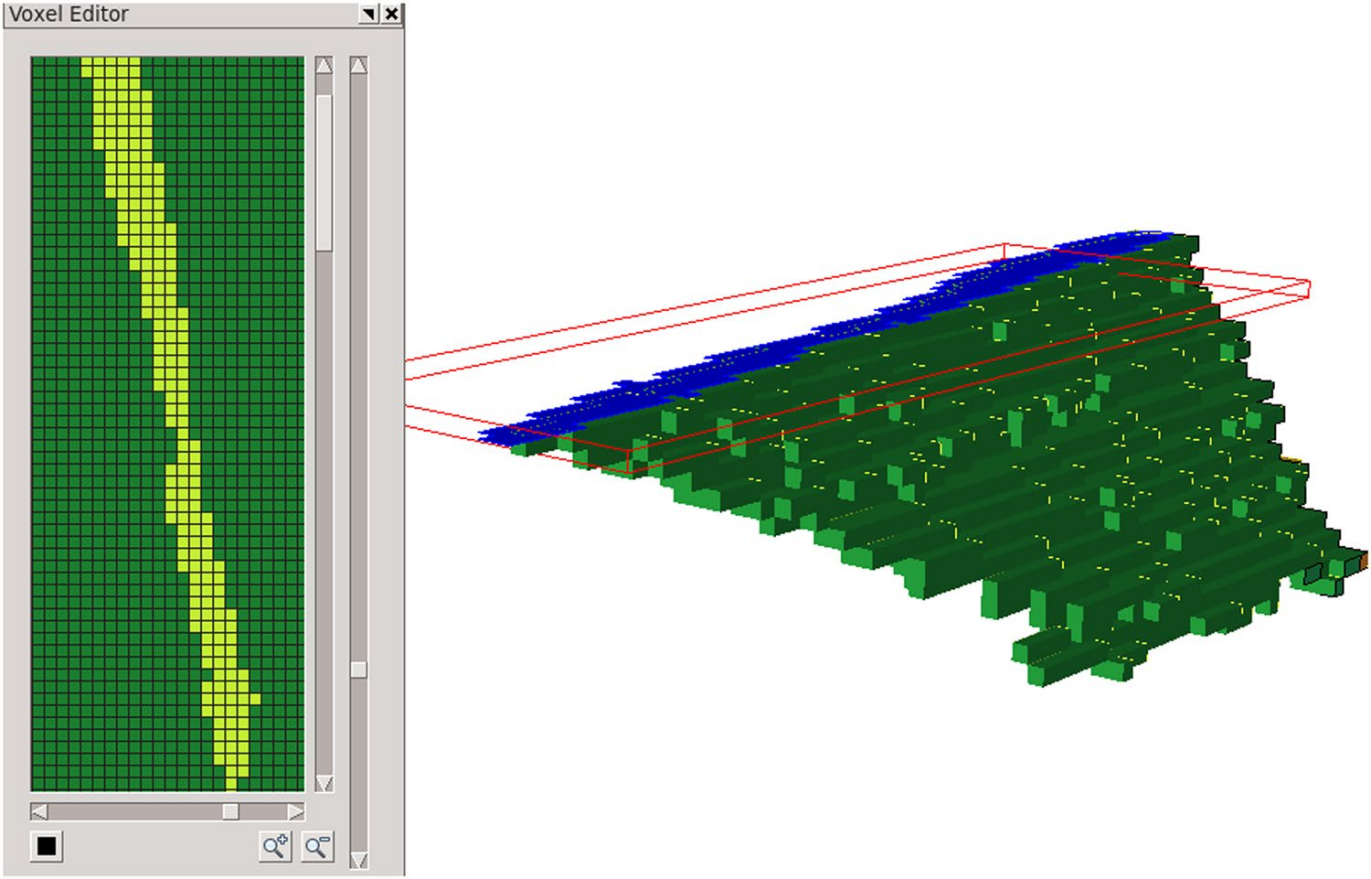
Cut plane through the grain number 105. Twin grains are as thin as one voxel at times within the voxelized 3D dataset.

**Fig. 14:**
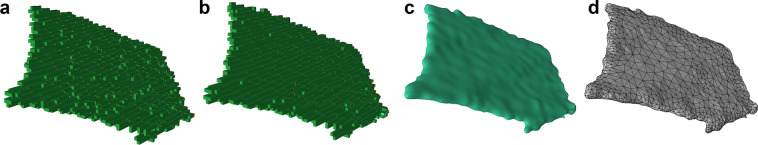
Grain number 105 as it passes through the modeling process. (**a**) Initial voxel-level grain representation. (**b**) the voxel data after artifacts were removed. (**c**) geometric representation of the grain and, (**d**) the corresponding discretization.

**Fig. 15:**
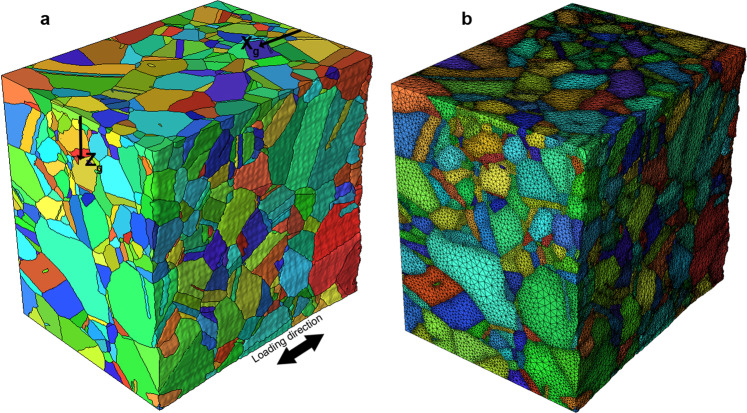
Modeling procedure using Simmetrix’ software suite. (**a**) Geometric representation of the In718 RVE created from the serial-sectioning voxel dataset. (**a**) Volumetric discretization of the In718 RVE.

**Fig. 16:**
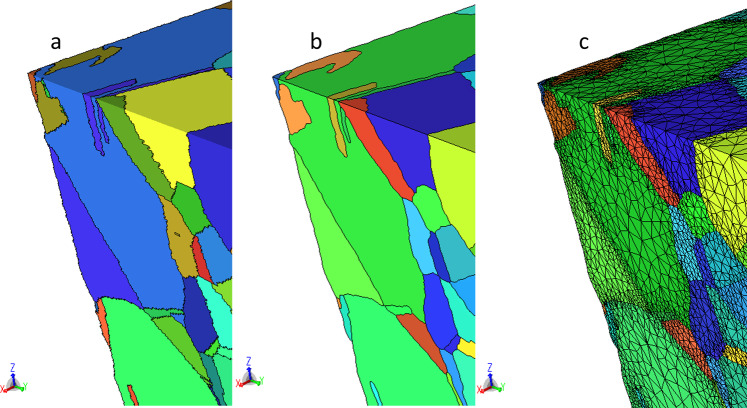
Close-up view of the RVE representation. (**a**) Voxelized 3D dataset. (**b**) Geometric model. (**c**) Volumetric mesh.

For the In718 RVE, features smaller than 50 connected voxels were removed followed by an erosion/dilation step using a 3x3x3 block structuring element. Many of the grains in this dataset were rather thin (often only one or two voxels thick). These were not processed using the erosion/dilation algorithm since it would completely remove them from the data. Figure 13 shows a twin grain (identified as grain ID 105 in the DREAM3D dataset) with a cut plane showing that the grain is at times only one voxel thick. Figure 14 shows pictures of that grain as it goes through the reconstruction and meshing process, from the original voxel dataset to the voxel structure after artifacts were removed, followed by the smoothed grain surface representation and the associated volumetric mesh. Figure 15(a) shows a view of the In718 RVE geometric model that was constructed from the voxel dataset. The corresponding mesh shown in Fig. 15(b) was created by assigning a mesh size attribute with value 0.3 μm on all grain boundaries, resulting in a total of 16.7 million tetrahedral elements. A close-up view of the In718 RVE model evolution from a voxel level representation to a geometric reconstruction and an associated volumetric mesh is provided in Fig. 16.

## Data Records

### SEM Images

The raw SEM images used for HR-DIC calculations are 16-bit tiff images and can be downloaded from the DRYAD repository^29^. The nomenclature and location of the images are detailed in Fig. 2. The image origin is located for the pixels within the tiff images, which is set as the first top-left pixel, with the x-tiff (X*g*) proceeding to the right, and y-tiff (Z_*g*_) proceeding down the image.

### Full-field measurements from HR-DIC

The HR-DIC data are provided as 32-bit tiff images. The images can be viewed using software such as ImageJ. The values at each pixel in the images are the quantitative physical value of the displayed full fields given in unit length for the strain field *ε*_*xx*_, in pixels for the in-plane slip amplitude, and in radians for the in-plane slip direction. We refer the reader to^10,12^ for more information about the physical meaning of these quantities. The data can be downloaded from the DRYAD repository^29^. Adjacent pixels in all maps represent a physical distance of 100.34 nm. The values in the in-plane slip amplitude maps are given in pixels, where one pixel represents a shearing/displacement induced by slip of 33.45 nm. The image origin is located for the pixels within the tiff images, which is set as the first top-left pixel, with the x-tiff (X_*g*_) proceeding to the right, and y-tiff (Z_*g*_) proceeding down the image.

### 2D Crystallographic orientation data

The IPF maps along the loading direction X (see Fig. 4) from the raw EBSD measurements and after cleaning and distortion (to match the DIC full field maps) are provided in a tiff format in a RGB color type and labeled as IPF_Raw X.tif and IPF_DistordedDIC_X.tif, respectively. The IPF map along the transverse direction Zg (see Fig. 4) is also provided and labeled as IPF_Raw_Z.tif. The raw crystallographic orientation data (Euler angle and grain structure) are provided in the .osc and .ang format and labeled as Euler_Orientation_Raw.osc and Euler_Orientation_Raw.ang. The .osc and .ang format are labeled as Euler Orientation Raw.osc and Euler Orientation Raw.ang. The .osc format can be opened using the TSL software from EDAX. The .ang format is a ASCII text format where the first three columns displayed the Euler angles *ϕ*_1_, Φ and *ϕ*_2_ in radians. Columns 4 and 5 display the coordinate X_*g*_ and Z_*g*_, respectively, for a given measurement point. It should be noted that the actual data column 5 in the .ang file are incorrectly labeled Y. Again, the label Y in column 5 corresponds to the global direction Z_*g*_. Columns 7 and 8 display the image quality (IQ) and confidence index (CI) associated with the collected Kikuchi patterns.

### 3D Dataset - voxelized structure

The raw Kikuchi patterns obtained from EBSD measurement are provided in the .up2 binary file format for each EBSD slice from the TriBeam tomography measurement, these files can be opened using EMsoft dictionary indexing^30,31^, EMSphInx^32^ spherical pattern reindexing or with the commercial EDAX or Oxford EBSD analysis software. The data can be downloaded from the DRYAD repository^29^; however, the up2 datafiles are each 3.26 GB resulting in a large approximately 1.71 TB download. The reconstructed DREAM3D dataset can be downloaded independently from DRYAD^29^ for the interested reader who does not require the raw up2 EBSD patterns. The indexed data from each slice are also provided in the EMsoft HDF .h5 formatted files. The BSE and SEM images obtained after each slice during the TriBeam experiment are provided in 8-bit tiff images. The reconstructed 3D data with the distortion correction applied is provided in the .dream3d file (h5 structure). The Hierarchical Data Format version 5 (h5), is an open source file format that supports large, complex, heterogeneous data. H5 file uses a “file directory” like structure that allows one to organize data within the h5 file in many different structured ways, as one might do in a standard computer file system. The h5 format also allows for embedding of metadata making it self-describing. The associated .xdmf file provides the container data descriptions. Such files can be opened using the open-source software Paraview (https://www.paraview.org/) for visualization. For more information about the different conventions used to store the 3D EBSD data, we encourage the reader to consult the following references^18,19^.

### 3D Dataset - meshed structures

The mesh generated with XtalMesh is provided in two file formats, .inp and .vtk. The .inp file, labelled XtalMesh.inp, is used as input into ABAQUS. The .inp file format is standard to ABAQUS and is an ASCII data file that consists of a series of ABAQUS keyword and data lines. In it, all nodes, elements, and element sets of the mesh are defined. There, the mesh is represented by ten-node tetrahedral elements (C3D10). The .vtk file, labelled XtalMesh.vtk, is a binary Visualization Toolkit (VTK) datafile provided for ease of visualization and analysis of the mesh using ParaView by Kitware. There, the mesh is represented by four-node tetrahedral elements (C3D4) to reduce system load during visualization.

The mesh generated with the software suite from Simmetrix (SimModeler Voxel), is provided in an Ansys^33^ input deck. The input deck contains the following APDL (Ansys Parametric Design Language) files: run_Model.mac, contains all the calls to load the mesh, assign material properties, define node components, orient each grain according to the average Euler angles from the EBSD measurement and, for example, assign boundary conditions. The file In718RVE.cdb contains element connectivity and nodal coordinates of the entire RVE model, assignWPCSYS.mac creates coordinate systems using Euler angles (each coordinate system ID is linked to the element coordinate system defined for each grain), EulerAngles.txt contains the averaged Euler angles (available in the .dream3d file) for each grain in the reconstructed microstructure, In718RVE_pd.cdb assigns element coordinate systems (ESYS command in APDL) to each element cluster that defines a grain and creates node components on each side of the RVE for an easy assignment of boundary conditions, assignMatProp.mac assigns material properties (cubic elasticity constitutive model^2^) to all grains, and In718RVE_ss.cdb performs the finite element solution. The finite element model setup presented above was tested to make sure a solution can be obtained with the Ansys solver.

## Technical Validation

### HR-DIC Measurements

Initial validation of the high-resolution strain fields obtained by HR-DIC is achieved by directly comparing the calculated average *ε*_*xx*_ strain along the loading direction over the representative region of interest with the measured macroscopic strain along the gauge length of the specimen. HR-DIC and macroscopic strains are reported in Table 1.

**Table 1:** Comparison between macroscopic total strain and average *ε*_*xx*_ from the HR-DIC measurements.

Load step	E1	E2	E3	E4
Macroscopic strain	0.69%	0.91%	1.23%	1.83%
Average *ε*_*xx*_ strain from HR-DIC	0.68% (1.5%)	0.87% (4.4%)	1.16% (5.7%)	1.70% (7.1%)

The absolute difference between average *ε*_*xx*_ from HR-DIC measurements and macroscopic strain is given in parentheses.

**Fig. 17:**
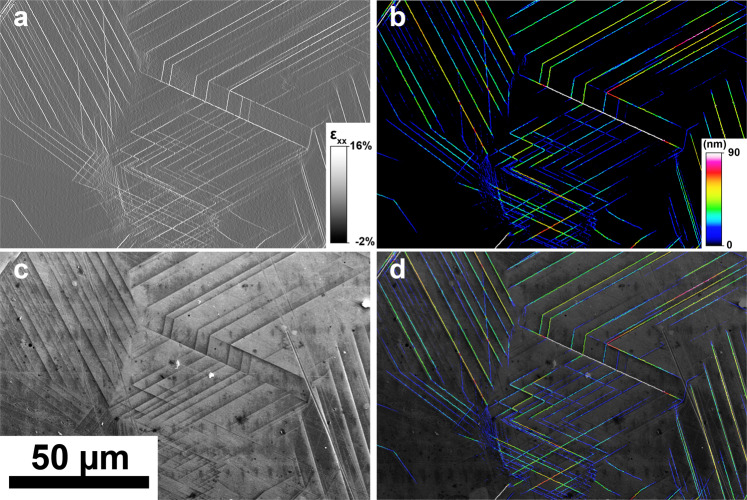
Experimental validation by slip trace imaging. (**a**) HR-DIC strain field *ε*_*xx*_ for a reduced region of interest. Intense strain bands show the location of slip events. (**b**) The associated in-plane intensity of slip given in nanometers. (**c**) BSE image for the same region of interest. BSE imaging is an established SEM modality to image slip events. (**d**) Overlap of the HR-DIC measurement (slip intensity) with the BSE image. Perfect agreement is observed between BSE imaging and HR-DIC measurement.

Good agreement is observed between the macroscoscopic total strain and average *ε*_*xx*_ from the HR-DIC measurements. The differences in percent of the total deformation between the two measurements are provided in brackets in Table 1 for the different load steps. Further validation of the HR-DIC measurements is provided by direct comparison of the HR-DIC fields to high resolution BSE images and displayed in Fig. 17 for a reduced region of interest. BSE imaging is a common measurement technique for capturing the location and existence of slip events at sample surfaces. Every single slip event is captured by the HR-DIC measurement and their locations are identical to those identified in the BSE images. The intensity of the strain concentration and in-plane slip obtained by the HR-DIC measurement and using the Heaviside-DIC method was previously validated^10^. The intensity of slip is obtained for each single slip trace with high spatial (less than 33 nm) and amplitude resolution (less than 10 nm).

### 3D Microstructure

**Fig. 18:**
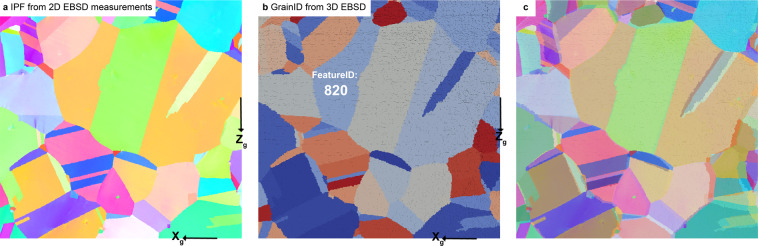
Experimental validation of the grain boundary locations. (**a**) Inverse pole figure map for a reduced region of interest obtained from the EBSD measurement performed at the surface of the specimen before TriBeam serial sectioning. (**b**) FeatureID maps at the surface of the 3d dataset of the same region of interest obtained after TriBeam serial sectioning and 3D reconstruction. (**c**) Overlap of (**a** and **b**). Very good agreement is obtained between the grain morphology from conventional EBSD measurement and TriBeam serial sectioning.

To validate the structure of the grains in the 3D dataset, the grain structure at the free surface of the reconstructed 3D dataset is extracted and compared to the conventional EBSD measurement performed at the free surface of the specimen, described in the Section Surface Crystallographic Orientation Measurements. The inverse pole figure map in Fig. 18(a) of a reduced region of interest obtained from the surface EBSD measurement describes the grain structure at the surface of the specimen. The associated grain ID map obtained from the reconstructed 2D dataset is presented in Fig. 18(b). An overlap of the two maps are presented in Fig. 18(c) showing that both measurements display identical grain structure. However a slight difference in the location of the grain boundaries is observed. As a consequence, 7% of the total surface area is observed to not match (within 1 pixel tolerance) between the free surface obtained from the reconstructed 3D dataset and the free surface obtained from conventional surface measurement.

**Fig. 19:**
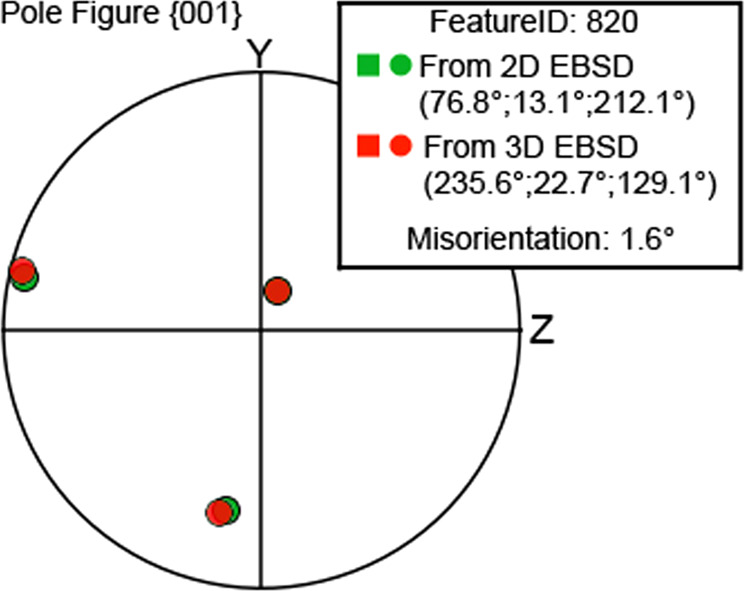
Experimental validation of the crystallographic orientation. Crystallographic orientation as displayed in the pole figure 001 of the grain labelled 820 in Fig. 18 from conventional EBSD measurement at the surface of the specimen and TriBeam serial sectioning. A minimal misorientation is observed between the two experimental measurements.

For validation of the crystallographic orientation, a direct comparison of the orientation obtained between conventional surface measurement and TriBeam serial sectioning measurements are performed for the grain labeled “FeatureID 820” in Fig. 18(b). The average orientations obtained at the surface of the grain are displayed in Fig. 19 using pole figure. An insignificant misorientation of 1.6° between the two measurements is observed.

### Meshed dataset

**Fig. 20:**
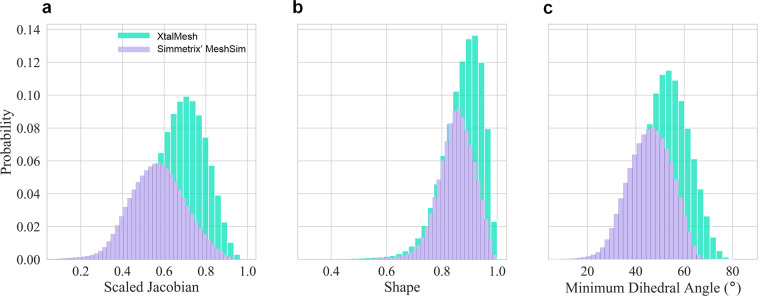
Mesh quality statistics for the meshed structure generated with XtalMesh and Simmetrix’ MeshSim. (**a**) Metrics include scaled Jacobian, (**b**) shape, and (**c**) minimum dihedral angle as defined by “The Verdict Library Reference Manual”^34^.

The generated meshes are evaluated using the tetrahedral quality metrics, that include the scaled Jacobian, shape, and minimum dihedral angle as defined by Stimpson *et al*. in “The Verdict Library Reference Manual”^34^. Each quality metric represents a unique mathematical measure of an element’s level of distortion from a perfect equilateral tetrahedron, and as no one metric is universally applied, multiple have been provided. Mesh quality statistics are provided in Fig. 20 for both the generated mesh using XtalMesh and Simmetrix’ MeshSim, which each displayed high mesh quality metrics.

## Usage Notes

We hope this article will encourage other research groups to share their 3D datasets as a growing collection of multi-modal datasets would serve as a unique benchmark for the design of new microstructures or prediction of mechanical properties for structural materials. This set of high-resolution images provides opportunities to evaluate DIC methods and compare with the DIC method presented here. This set also was contributed to the effort of the Society of Experimental Mechanics DIC challenge (www.sem.org/dic-challenge/) by providing recent high resolution scanning electron microscope images for DIC.

The 3D dataset and the various imaging modalities are also shared on the BisQue platform^35^ at: https://bisque.ece.ucsb.edu/client_service/view?resource=https://bisque.ece.ucsb.edu/data_service/00-Go3oRQiehaBUzET2cBAeug. In BisQue, the data provenance can be explored and DREAM.3D pipelines can be modified and executed to perform other types of analysis on the dataset.

The TriBeam microscope used to collect the 3D dataset is a prototype system developed at UC Santa Barbara. However, an updated and commercially available instrument is available from Thermo Fisher Scientific^36^. More broadly, data of this modality is available via serial sectioning methods^37^ or via X-ray methods^38^, which are especially well suited for *in-situ* measurements.

In addition, the multi-modal dataset can be used for direct comparison to crystal plasticity simulations including Fast-Fourier transform based calculations (FFT - operating on the voxelized data directly) or by finite element calculations (FE - operating on a meshed version of the data). Two full 3D volume meshes suitable for FE calculations have been generated from the 3D microstructural dataset collected by TriBeam and are provided. We encourage other researchers to use it for calculation of inter- and intragranular mechanical fields, and for the study of various deformation mechanisms.

## Data Availability

Multi-modal datasets are of great interest for evaluating the dataset-merging procedures for distortion correction and for correlative measurements analysis tools. The distortion correction algorithms and data analysis tools used in this study can be found at https://github.com/charpagne. Reindexing of the EBSD data with the dictionary indexing approach was performed with EMsoft version 4.2, the latest version of the code is available at https://github.com/EMsoft-org/EMsoft. The 3D voxelized dataset has been reconstructed using DREAM.3D, a software publicly available on GitHub (https://github.com/BlueQuartzSoftware/DREAM3D) or in the following link for direct download (http://dream3d.bluequartz.net/Download/. One version of a mesh structure was created with XtalMesh^21^, a publicly available code on GitHub (https://github.com/jonathanhestroffer/XtalMesh). The second version was created with the software suite from Simmetrix (SimModeler Voxel), a commercially available software (http://www.simmetrix.com/). Digital image correlation were performed using the software Xcorrel HDIC. The authors should be contacted for further information on this in-house software.
